# White matter microstructural changes in adolescent anorexia nervosa including an exploratory longitudinal study

**DOI:** 10.1016/j.nicl.2016.04.002

**Published:** 2016-04-12

**Authors:** Katja Vogel, Inge Timmers, Vinod Kumar, Thomas Nickl-Jockschat, Matteo Bastiani, Alard Roebroek, Beate Herpertz-Dahlmann, Kerstin Konrad, Rainer Goebel, Jochen Seitz

**Affiliations:** a Department of Child and Adolescent Psychiatry, Psychotherapy and Psychosomatics, University Hospital, RWTH Aachen University, Neuenhofer Weg 21, 52074 Aachen, Germany; b Department of Cognitive Neuroscience, Faculty of Psychology and Neuroscience, Maastricht University, P.O. Box 616, 6200 MD, Maastricht, The Netherlands; c Department of Psychiatry, Psychotherapy and Psychosomatics, University Hospital, RWTH Aachen University, Pauwelsstrasse 30, 52074 Aachen, Germany; d JARA-Translational Brain Medicine (The Jülich Aachen Research Alliance), Forschungszentrum Jülich GmbH, 52425 Jülich, Germany; e Oxford Centre for Functional MRI of the Brain, John Radcliffe Hospital, Oxford OX3 9DU, United Kingdom

**Keywords:** Anorexia nervosa, Adolescence, DTI, TBSS, Fractional anisotropy

## Abstract

**Background:**

Anorexia nervosa (AN) often begins in adolescence, however, the understanding of the underlying pathophysiology at this developmentally important age is scarce, impeding early interventions. We used diffusion tensor imaging (DTI) to investigate microstructural white matter (WM) brain changes including an experimental longitudinal follow-up.

**Methods:**

We acquired whole brain diffusion-weighted brain scans of 22 adolescent female hospitalized patients with AN at admission and nine patients longitudinally at discharge after weight rehabilitation. Patients (10–18 years) were compared to 21 typically developing controls (TD). Tract-based spatial statistics (TBSS) were applied to compare fractional anisotropy (FA) across groups and time points. Associations between average FA values of the global WM skeleton and weight as well as illness duration parameters were analyzed by multiple linear regression.

**Results:**

We observed increased FA in bilateral frontal, parietal and temporal areas in AN patients at admission compared to TD. Higher FA of the global WM skeleton at admission was associated with faster weight loss prior to admission. Exploratory longitudinal analysis showed this FA increase to be partially normalized after weight rehabilitation.

**Conclusions:**

Our findings reveal a markedly different pattern of WM microstructural changes in adolescent AN compared to most previous results in adult AN. This could signify a different susceptibility and reaction to semi-starvation in the still developing brain of adolescents or a time-dependent pathomechanism differing with extend of chronicity. Higher FA at admission in adolescents with AN could point to WM fibers being packed together more closely.

## Highlights

• Semi-starvation in anorexia nervosa (AN) leads to dramatic neuroanatomical changes.
• We investigated microstructural white matter brain changes in adolescents with AN.
• Fractional anisotropy was increased, differing from previous results in adults.
• This difference could be related to the developing brain or shorter illness duration.
• Experimental longitudinal follow-up showed partial normalization upon weight gain.

## 1 Introduction

Anorexia nervosa (AN) is the third-most common chronic disease in adolescents (Nicholls and Viner, 2005). It is characterized by distorted body image perception, extreme fear of weight gain and a restriction of energy intake leading to excessive weight loss (American Psychiatric Association, 2013).

The high-risk age group affected by AN is females between 15 to 19 years (Smink et al., 2012); 40% of disease onsets occur in this age range (Herpertz-Dahlmann et al., 2011). Almost 1 in 100 women will be affected by AN (Bulik et al., 2006). It is commonly suspected that AN has a multifactorial genesis with influences of genetic, prenatal and perinatal factors, hormonal changes during puberty, cultural influences and stressful life events as well as brain development-related factors (Herpertz-Dahlmann, 2015). Volumetric brain changes concerning grey matter (GM) and white matter (WM) are frequently found; however, these changes remain incompletely understood. Low body-weight seems to be associated with reduced GM and WM volume (Katzman et al., 1996, Seitz et al., 2015). Our recent meta-analysis revealed an average 3.2% reduction of GM and a 4.0% reduction of WM in adults with AN, whereas adolescents exhibited a 10.8% decrease in GM and a 3.1% decrease in WM (Seitz et al., 2014), suggesting that brain changes in adolescents with AN might differ from those in adults. The effects of brain volume changes in AN are largely reversible in those who exhibit weight recovery (King et al., 2014, Mainz et al., 2012); however, it remains unclear whether the restitution process is complete as longitudinal studies with longer follow-up periods are lacking. Importantly, WM volume reduction in acute AN seems to predispose patients to a lack of weight recovery at one-year follow-up (Seitz et al., 2015).

Diffusion tensor imaging (DTI) measures the diffusion of water molecules in the brain and uses the fact that diffusion is affected by tissue microstructure. Main indices of DTI have been linked to myelination, axon density, axon diameter, membrane permeability and the way in which axons are laid out within the voxel (Jones et al., 2013).

Despite the importance of adolescence in the pathogenesis of AN, only two research groups examined WM microstructure with DTI in adolescent patients with AN: Frank et al. (Frank et al., 2013) and Travis et al. (Travis et al., 2015). Frank et al. reported *decreased* fractional anisotropy (FA, a marker for the directedness of diffusion) in several brain regions compared to typically developing controls (TD). They also reported *increased* FA in different brain regions, however, they did not comment on its potential significance. Travis et al. examined nine bilateral cerebral tracts and eight callosal white matter tracts of AN patients. They found *decreased* FA in four of 26 WM tracts (right anterior superior longitudinal fasciculus, bilateral fimbria-fornix, motor subdivision of corpus callosum) and *increased* FA in two of 26 WM tracts (right anterior thalamic radiation, left anterior superior longitudinal fasciculus) compared to TD. R1, an index of myelin content, was found to be decreased in 11 of 26 WM tracts in AN patients. They concluded reduced FA in combination with reduced R1 to be due to reduced myelin content, however, for the majority of tracts the group differences did not occur in the same tract. R1 was also reduced in regions with increased FA, hence they hypothesized increased FA likely to be related to alterations in myelin leading to increased fiber coherence from axonal loss.

Other research groups examined WM microstructure in adult female AN patients in contrast to healthy controls with more homogenous results compared to those in adolescents (Frieling et al., 2012, Hayes et al., 2015, Kazlouski et al., 2011, Nagahara et al., 2014, Via et al., 2014): FA was mainly *reduced* in several brain regions of patients with AN. Hayes found reduced axial diffusivity (AD, parallel to the main direction of diffusion). Mean diffusivity (MD, total diffusion in all directions, often found to be inversely related to FA) and radial diffusivity (RD, diffusion perpendicular to the main direction of diffusion) were mainly *increased*. This is in line with a reduction in the density of axons or demyelination (Nagahara et al., 2014, Via et al., 2014), however, it should be cautioned that a direct interpretation of DTI parameters might be too simplistic (Jones et al., 2013).

In adult patients with AN a positive correlation between FA and current body mass index (BMI) (Kazlouski et al., 2011, Nagahara et al., 2014, Yau et al., 2013) as well as lowest lifetime BMI (Yau et al., 2013) was found, supporting that major changes in WM integrity are linked to a more severe and chronic illness course.

Shott et al. studied recovered adult patients and showed a regional *decrease* of FA similar to acutely ill patients (Shott et al., 2015). Reduced FA correlated with longer illness duration, indicating chronic alteration in recovered patients. However, in another study of former patients with AN Yau et al. reported opposing findings of *decreased* MD. They interpreted these findings as potentially preexisting increases and also found them to be linked to perfectionism and increased cognitive control, factors predisposing for AN.

In summary, most findings in adults with AN point to *reduced* FA and *increased* MD which are linked to illness severity and illness duration, while findings in adolescents are scarce and ambivalent.

Our study used DTI to examine adolescent patients when they presented with acute starvation (admission) compared to TD. In an exploratory longitudinal analysis, a subsample of patients was also scanned for a second time at the end of their weight rehabilitation. Our study focused on adolescent female patients with AN, thus studying the high-risk age group to further our understanding of (patho-) physiological processes in the developing brain following semi-starvation. By studying adolescent patients, typically at the beginning of the disease, pathologies can be expected to be less altered by chronic processes and secondary or compensatory mechanisms compared to adults with AN. We hypothesized that patients would exhibit altered FA and MD in acute starvation relative to TD. We expected our findings in adolescent AN to differ from those in previous studies of adult patients affected at a later stage of brain development. Furthermore, we anticipated partial normalization of WM microstructure upon weight rehabilitation.

## 2 Methods

### 2.1 Participants

Patients were recruited in the Department for Child and Adolescent Psychiatry of RWTH Aachen University Hospital from July 2009 to November 2012. Female patients aged 10–18 years with the diagnosis anorexia nervosa (DSM-IV 307.1) and BMI ≤ 10th age-percentile at the beginning of the inpatient treatment were included. Exclusion criteria were as follows: drug dependency, history of or current psychosis, inability to communicate in the German language or general exclusion criteria for MRI studies, such as irremovable metal objects in the body.

Age and sex matched healthy controls with normal body weight were recruited in local schools in 2012. Subjects with a history of psychiatric disorder, eating disorder, weight outside the 10th or 90th percentile, non-removable metal implants, lack of German language ability or pregnancy were not eligible for the control group.

All subjects and their parents provided informed written consent. The ethical committee of the Medical Faculty of RWTH Aachen University approved the study, which was conducted in accordance with the principles of the Helsinki declaration.

The presence of eating disorders or other psychiatric comorbidities in patients was assessed by standardized clinical interviews by an experienced clinician according to DSM IV using the adolescent “Schedule for Affective Disorders and Schizophrenia” (K-SADS). In addition, we collected the following clinical parameters: illness duration prior to admission, weight loss prior to admission, rate of weight loss, age at admission, BMI-SDS at admission, weight gain during therapy, duration of therapy, age at discharge and BMI-SDS at discharge.

A total of 22 female patients participated in the study. 22 patients were scanned at the beginning of the inpatient treatment (admission). Nine patients were also scanned at the end of weight rehabilitation (discharge). 21 age-matched TD also underwent DTI scans.

There were 19 AN patients with restricting subtype and three with binge-eating/purging subtype. Major depression was diagnosed in one patient and anxiety disorder in two patients and combined depression and anxiety disorder in one patient. One of these patients took diazepam at admission, and one patient took olanzapine at discharge. All other patients were medication-free. For patient and TD characteristics please cf. Table 1.

TD were assessed once, their age did not significantly differ from patients at admission or discharge (all p > 0.05) (cf. Table 1).

### 2.2 Image acquisition

Scanning was performed on a 3-Tesla Siemens Trio MR scanner in the RWTH Aachen University Hospital. Data were obtained using two different protocols differing in one parameter: 16 patients at admission, nine patients at discharge and 10 TD received protocol 1, the remaining participants received protocol 2. Both used diffusion-weighted double spin-echo planar imaging (EPI) sequence, repetition time 9400 ms, echo time 94 ms, and b-value = 1000 s/mm^2^ with 30 gradient directions matched in both protocols. The first protocol used 2 × 2-mm resolution and 65 axial slices with 2-mm thickness (protocol 1, isovoxel). The second protocol used 2 × 2-mm resolution with 34 axial slices with 3.5-mm thickness and a 10% gap (protocol 2, non-isovoxel). Two b = 0 images (T2-weighted) were acquired for each subject. Both protocols were acquired twice and the results were averaged.

### 2.3 Image analysis

Diffusion data were preprocessed to correct for bulk motion and eddy current-induced distortions (Andersson et al., 2003) using *topup* and *eddy* as implemented in FSL 5.0.6 (Smith et al., 2004) and corrected for B-matrix rotation following subject motion. Diffusion tensors were fitted to the acquired data by linear regression using a least-square minimization approach (*dtifit*), and MD, FA, AD and RD maps were computed using FSL 5.0.6 (Smith et al., 2004).

The data were processed and analyzed with FSL 5.0.6 using tract-based spatial statistics (TBSS) (Smith et al., 2006). For this, individual brain FA maps were non-linearly projected onto the standard MNI-template (FNIRT). Next, a mean FA skeleton map was produced, representing the centers of all major white matter tracts (FA > 0.2). Per subject, the aligned FA data were projected onto this skeleton, and then compared between subjects. This tract-based analysis improves registration of WM tracts of different brains and does not require smoothing (Smith et al., 2007, Smith et al., 2006). MD, AD and RD data were projected onto the same skeleton. On the skeletonized data, permutation-based statistics were used to compare AN at admission and TD on a voxel-by-voxel level (using FSL's randomize: 5000 permutations). Threshold-Free Cluster Enhancement (TFCE) (Smith and Nichols, 2009) was used to correct for multiple comparison. Age and protocol type were entered as covariates. To rule out a potential confounding effect of protocol type, we repeated the above analysis with all patients scanned with protocol 1 only and protocol 2 only. To rule out an effect of medication or comorbid diagnosis, we repeated the analysis excluding these four patients. In our exploratory longitudinal study, we furthermore compared AN after weight recovery with TD using the same procedure as above.

To replicate our results with a different analyses software, we also reanalyzed the data with Statistical Parametric Mapping (SPM8) as described by Nickl-Jockschat et al. (Nickl-Jockschat et al., 2015), (cf. supplementary material). In brief we applied Unified Segmentation to the non-diffusion weighted B0 images of each data set. The resulting deformation fields were used on each individual FA map to transform them into MNI space. An 8 mm smoothing kernel was applied. TFCE as implied in the TFCE toolbox v73 (http://dbm.neuro.uni-jena.de/tfce/) for SPM was used for statistical analysis.

To specify the changes underlying alterations in FA, we defined all clusters indicating significant FA differences between AN at admission and the control group in the TBSS analysis as regions-of-interest (ROIs) and subsequently extracted the average diffusivity values (FA_roi, MD_roi, AD_roi and RD_roi) of all voxels in the respective ROIs. Additionally, we extracted the global averages of all major white matter tracts of the brain with FA > 0.2 (FA_global, MD_global, AD_global, RD_global). These average diffusivity values were used for further comparisons between the groups using two-way ANOVA correcting for age and protocol type using SPSS 22. To test for intra-individual longitudinal changes in the exploratory longitudinal study, we calculated a paired Student's *t*-test of average ROI diffusivity values of all patients with AN with longitudinal data at admission and discharge that were scanned with the identical protocol 1.

Routinely collected urine samples of patients were analyzed for their specific gravity and compared with norm values (1003–1030 g/l) (“Urine specific gravity”, 2014) to rule out dehydration as a cause for our findings. All urinary specific gravity values at admission and discharge were within the normal range. We correlated urinary specific gravity with the above average diffusivity values using Pearson's correlation correcting for age and protocol type. Urine specific gravity was not analyzed for TD.

To further investigate the factors influencing FA-changes at admission and discharge we entered the average FA_global at admission and at discharge as dependent variables in two separate multiple linear regression analyses. Independent variables for FA_global at admission were age at admission, BMI-SDS_admission, illness duration prior to admission, rate of weight loss (in parts of BMI-SDS/week) and protocol type. We repeated above analysis for the exploratory longitudinal study after weight rehabilitation. Independent variables for FA_global after weight rehabilitation were age at discharge, BMI-SDS_discharge, weight gain during therapy (in parts of BMI-SDS) and duration of treatment.

## 3 Results

### 3.1 Group comparison of FA, MD, RD and AD diffusivities between AN and TD on individual voxel-level using TBSS

**Supplementary Fig. 1:**
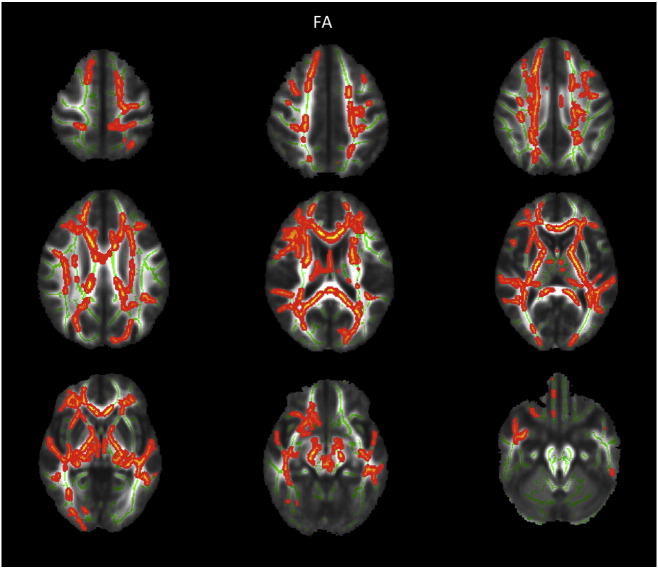
TBSS results comparing FA for adolescent AN at admission versus TD corrected for age. Only patients measured with the identical protocol one (iso-voxel) were entered in the analysis. Significant areas with AN > TD are marked in orange, AN < TD are marked in blue. Underlying WM skeleton marked in green. AN — anorexia nervosa FA — fractional anisotropy TD — typically developing controls.

**Supplementary Fig. 2:**
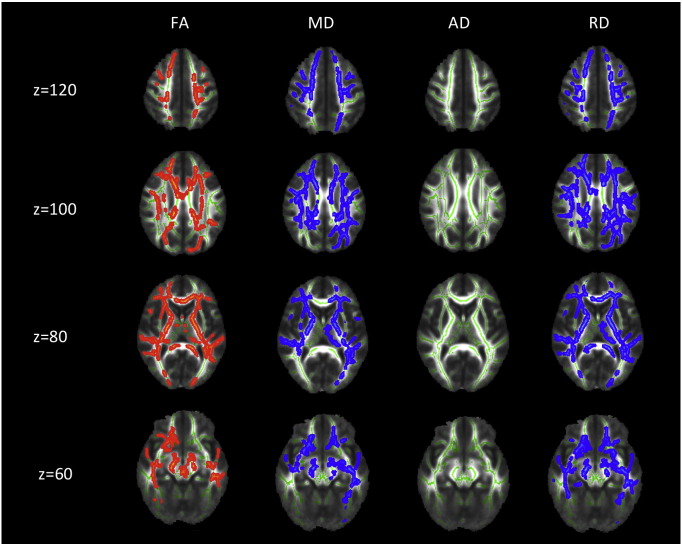
TBSS results comparing FA, MD, AD and RD for adolescent AN at admission versus TD corrected for age. Only patients measured with the identical protocol one (iso-voxel) were entered in the analysis. Significant areas with AN > TD are marked in orange, AN < TD are marked in blue. Underlying WM skeleton marked in green. AD — axial diffusivity AN_admission_ — anorexia nervosa patients at admission FA — fractional anisotropy MD — mean diffusivity RD — radial diffusivity TD — typically developing controls z — brain slice coordinate.

**Supplementary Fig. 3:**
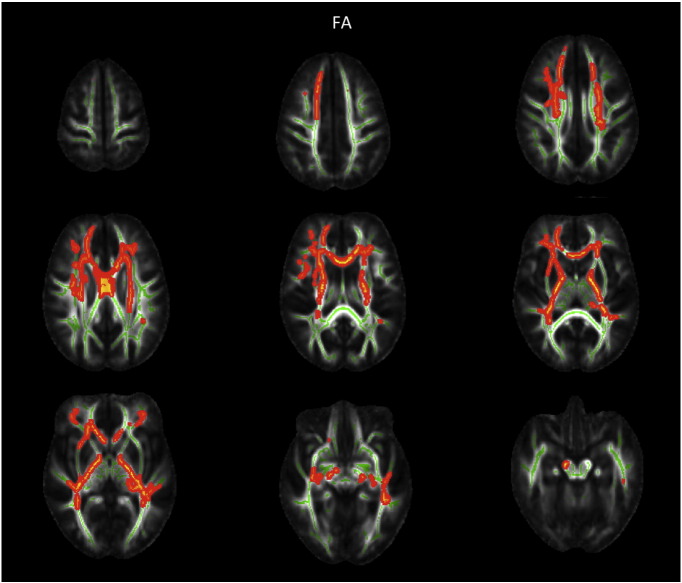
TBSS results using a different alignment strategy (DTI-TK) comparing FA for adolescent AN at admission versus TD corrected for age and protocol. Significant areas with AN > TD are marked in orange, AN < TD are marked in blue. Underlying WM skeleton marked in green. AN — anorexia nervosa FA — fractional anisotropy TD — typically developing controls

**Supplementary Fig. 4:**
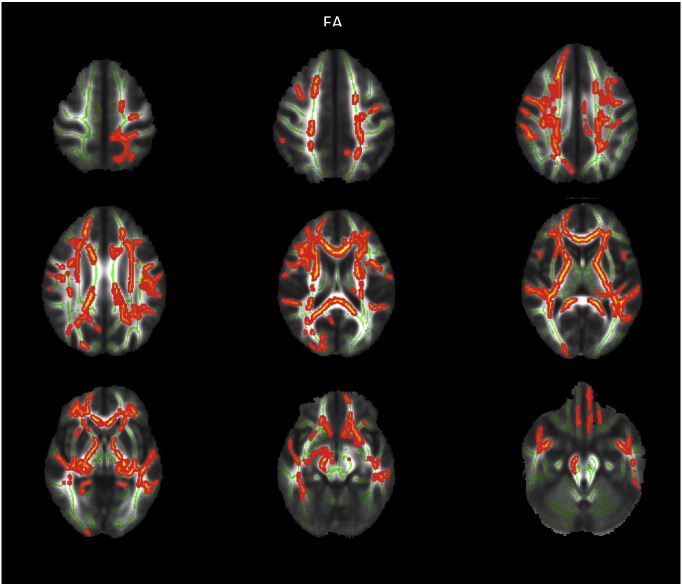
TBSS results excluding four patients with comorbidities and medication comparing FA for adolescent AN at admission versus TD corrected for age and protocol. Significant areas with AN > TD are marked in orange, AN < TD are marked in blue. Underlying WM skeleton marked in green. AN — anorexia nervosa FA — fractional anisotropy TD — typically developing controls.

**Fig. 1:**
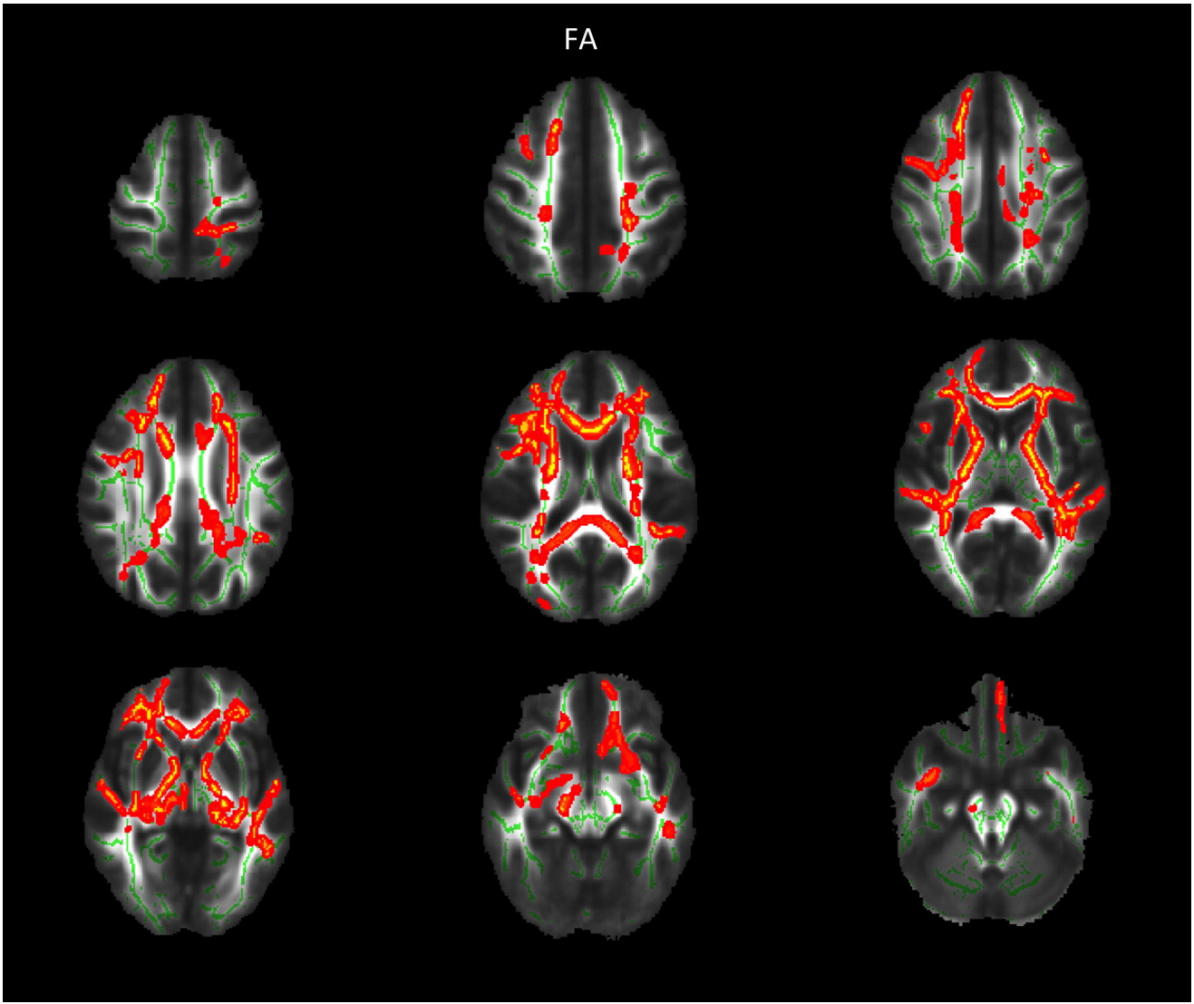
TBSS results comparing FA for adolescent AN at admission versus TD corrected for age and protocol. Significant areas with AN > TD are marked in orange. Underlying WM skeleton marked in green. AN — anorexia nervosa FA — fractional anisotropy TD — typically developing controls WM — white matter.

**Fig. 2:**
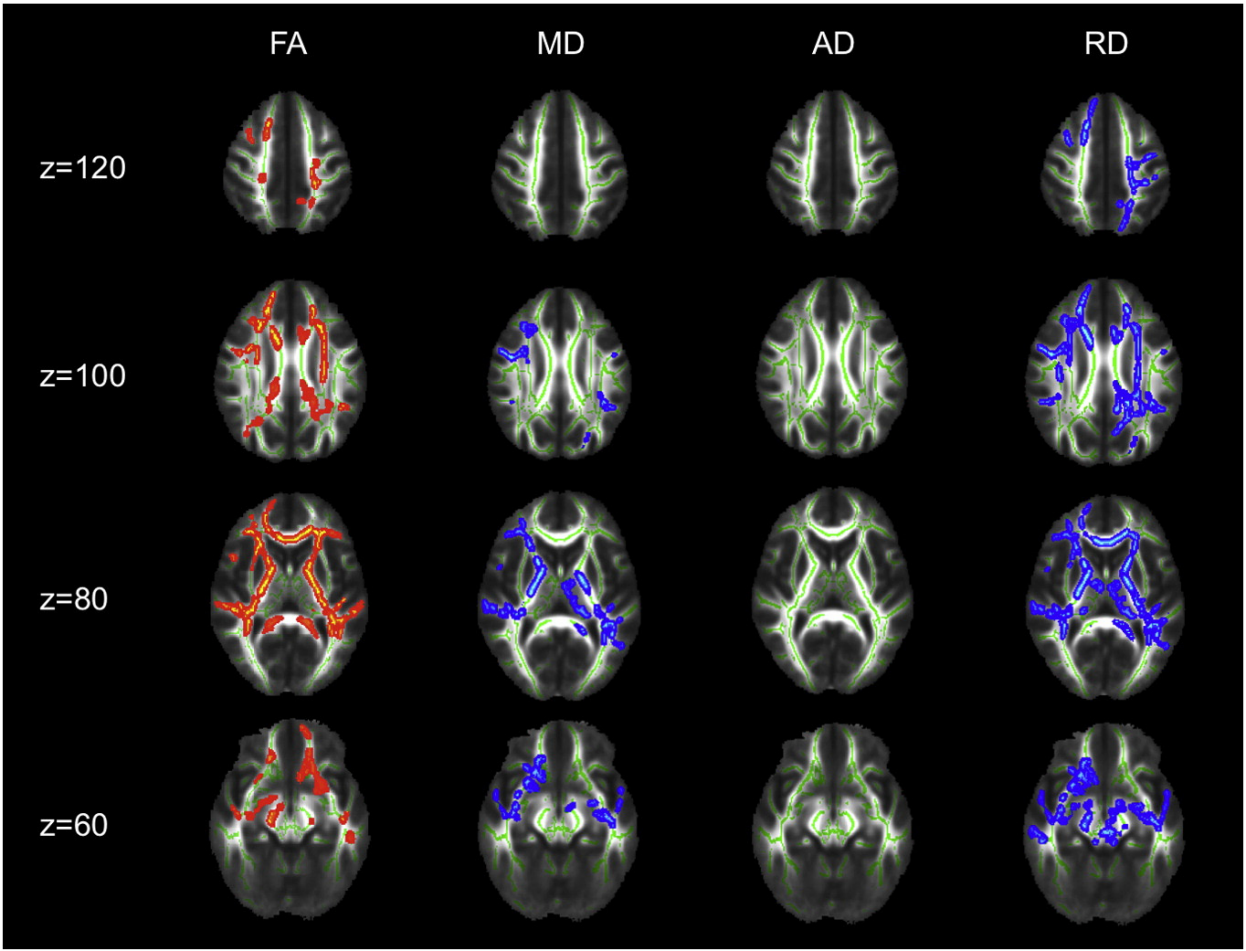
TBSS results comparing FA, MD, AD and RD for adolescent AN at admission versus TD. Significant areas with AN > TD are marked in orange, AN < TD are marked in blue. Underlying WM skeleton marked in green. AD — axial diffusivityAN — anorexia nervosaFA — fractional anisotropyMD — mean diffusivityRD — radial diffusivityTD — typically developing controlsz — brain slice coordinate.

Group comparison of FA revealed increased FA in AN patients at admission compared to TD in widespread frontal, parietal and temporal areas, including the bilateral superior region of corona radiata, corpus callosum anterior, anterior and posterior thalamic radiation, anterior and posterior limb of internal capsule as well as the left inferior longitudinal fasciculus (cf. Fig. 1). FA increase at admission was mainly due to reduced RD but not due to altered AD in those areas (cf. Fig. 2). Most areas with FA increase at admission also exhibited reduced MD. Repeating the above analysis including only participants scanned with protocol 1 resulted in similar results (cf. Supplementary Fig. 1, Supplementary Fig. 2), repeating with participants scanned with protocol 2 did not yield significant results, potentially due to smaller numbers. Repeating above analysis with a different alignment protocol (DTI-TK, (Bach et al., 2014)) yielded similar results (cf. supplementary Fig. 3). Repeating above analysis excluding four patients with depression, anxiety disorder or medication use yielded similar results (cf. supplementary Fig. 4). In the exploratory longitudinal study the comparison of FA of patients at discharge after weight rehabilitation with that of TD resulted in no individual region differing significantly between AN and TD in the cluster analysis.

**Supplementary Fig. 5:**
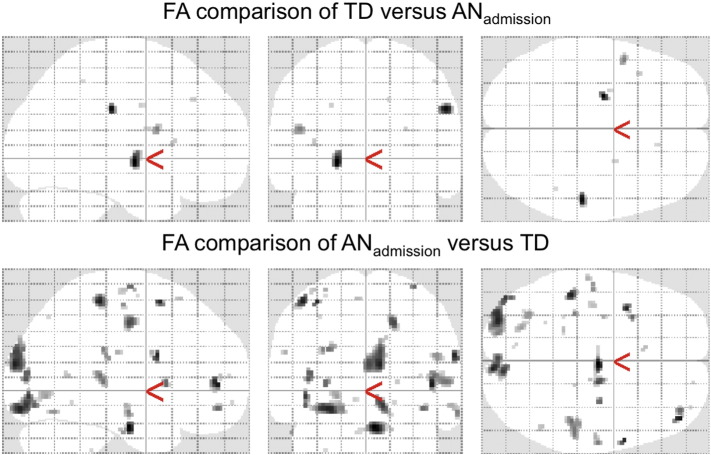
Alternative method (SPM) results comparing FA for adolescent AN at admission versus TD corrected for age and protocol. Top: Significant areas with TD > AN are marked in grey. Bottom: AN > TD are marked in grey. Uncorrected for multiple comparison. AN — anorexia nervosa AN_admission_ — anorexia nervosa patients at admission FA — fractional anisotropy TD — typically developing controls.

SPM analysis showed results pointing in the same direction as the TBSS-results described above (mostly increased FA and reduced MD in AN patients compared to TD), however, on a greatly reduced significance level (supplementary material, supplementary Table 1, supplementary Fig. 5).

### 3.2 Region of interest and average global diffusivity analysis

**Fig. 3:**
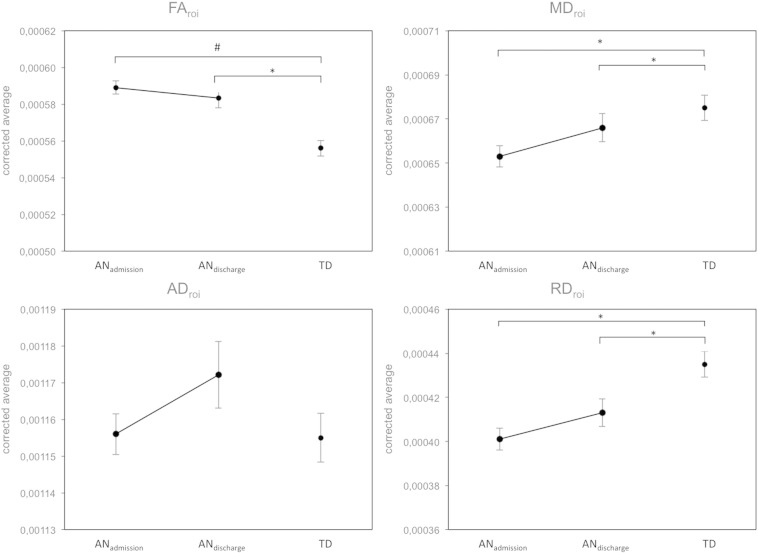
ANCOVA results comparing the region of interest differing in FA between AN_admission_, AN_discharge_ and TD for all diffusivity values corrected for age and protocol. AD — axial diffusivity AN_admission_ — anorexia nervosa patients at admission AN_discharge_ — anorexia nervosa patients at discharge FA — fractional anisotropy MD — mean diffusivity RD — radial diffusivity roi — region of interest TD — typically developing controls # — original contrast for defining roi * — p < 0.05.

Next, we extracted the average diffusivity values (FA_roi, MD_roi, AD_roi and RD_roi) for the above-mentioned regions significantly differing in FA between AN at admission and TD. ANOVA analysis comparing AN with TD was not performed for FA_roi for circularity reasons (the region had been defined using the same FA differences). However, ANOVA analysis showed significant reductions for MD and RD but not for AD (admission, protocol and age-corrected: MD_roi p = 0.002; RD_roi p < 0.001; AD_roi p = 0.359; cf. Fig. 3). In the exploratory longitudinal study diffusivity differences between patients and TD appeared partly reduced at discharge; however, FA remained significantly higher, and MD and RD remained significantly lower in patients with AN than in TD (discharge, protocol and age-corrected: FA_roi p = 0.005; MD_roi p = 0.002; RD_roi p = 0.048; AD_roi p = 0.563; cf. Fig. 3).

Paired *t*-tests analyzing these partial normalizations after weight restoration revealed a significant longitudinal reduction of FA_roi and a significant longitudinal increase of RD_roi (FA_roi, p = 0.008, RD_roi, p = 0.017). In contrast, the MD_roi increase exhibited a non-significant trend (MD_roi, p = 0.062), and AD_roi changes were not significant (AD_roi, p = 0.574).

**Supplementary Fig. 6:**
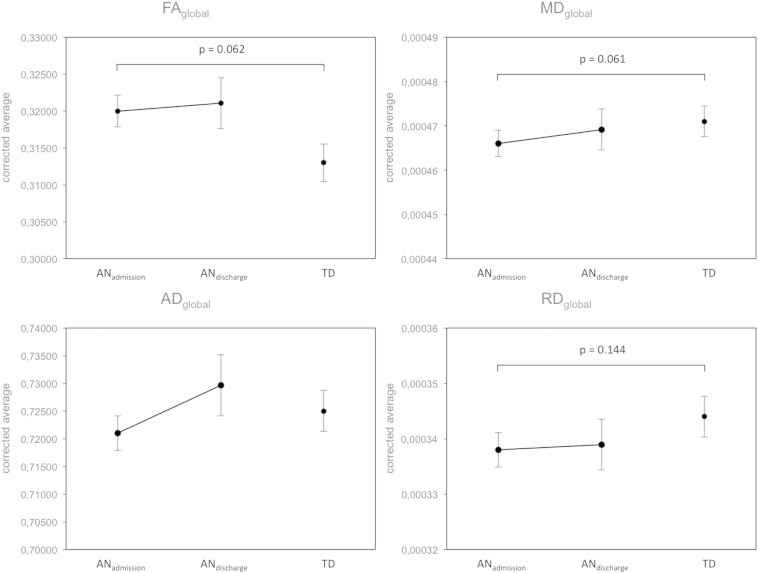
ANCOVA results comparing the global WM skeleton for all diffusivity values corrected for age and protocol. AD — axial diffusivity AN_admission_ — anorexia nervosa patients at admission AN_discharge_ — anorexia nervosa patients at discharge FA — fractional anisotropy MD — mean diffusivity.

Due to the large spatial extent of the above-mentioned regions of interest, we also extracted the average global WM diffusivity values (FA_global, MD_global, AD_global and RD_global) of the entire WM skeleton containing tracts with FA > 0.2. When comparing AN patients at admission with TD, FA_global and MD_global exhibited a trend in the same direction as the above findings (admission, protocol and age-corrected, FA_global p = 0.062, MD_global p = 0.061, AD_global p = 0.288, RD_global p = 0.144). The average global FA increase and MD and RD decrease were also partially normalized after weight rehabilitation but remained lower than in TD. All global changes in diffusivity exhibited the same directionality and general pattern as the region of interest above, however, without being statistically significant (cf. supplementary Fig. 6).

### 3.3 Clinical correlations

Urinary specific gravity was not correlated with any of the average diffusivity values (all p > 0.05).

FA_global at admission correlated with the rate of weight loss prior to admission in the multiple regression analysis with faster weight loss being associated with even higher FA values (cf. Table 2). Furthermore, in the exploratory longitudinal study, FA_global after weight recovery exhibited a positive correlation with standardized body mass index after weight recovery and with age. In contrast, FA_global after weight recovery exhibited a negative correlation with treatment duration in the same multiple regression analysis (cf. Table 2).

## 4 Discussion

To the best of our knowledge, this is the third study of WM microstructural changes in adolescent patients with AN and the first overall to include exploratory longitudinal data. Our findings suggest that adolescents with AN exhibit widespread changes in WM microstructure compared with TD including markedly increased FA. These alterations seem to be partially alleviated directly after weight recovery. Initial increase in global FA was associated with rapid weight loss in patients prior to admission. Thus, increased FA in adolescents with AN could be related to an acute, starvation-associated pathomechanism that is partially normalized after weight restoration. Our results of increased FA in adolescents differ distinctly from previous findings in adults with AN showing mainly reduced FA, but match partly the results in adolescents with AN showing increased FA (Frank et al., 2013, Travis et al., 2015). Differences to the latter adolescent studies with inconsistent findings could be explained by the different analysis techniques (TBSS versus VBM and individual tract based, highlighted by our own comparison with SPM as a second method), higher age (15.0 versus 15.4 and 16.6 years) and longer illness duration (12 versus 16.3 months). Potentially different pathophysiological mechanisms are also involved in the reaction of the developing brain to semi-starvation compared to mature brains of adults. Also, the different durations of semi-starvation in more recently ill adolescents versus more chronically affected adult patients with AN could play an important role.

In our study, increased FA at admission was associated with decreased MD and RD but not AD. RD was markedly decreased in the acute state of AN, whereas AD remained largely unchanged. This could signify closer packing of the myelinated axons in the WM (Beaulieu, 2002) which would obstruct diffusion orthogonal to the fibers. The packing could possibly stem 1) from a decrease of surrounding tissue volume, 2) from an increase in the fiber-diameter (e.g. from axonal swelling) (Beaulieu, 2002) or 3) from preexisting WM-fibers that exhibit more directionality (e.g. due to less crossing fibers) in patients than in TD. The first two explanations would be more likely consequences of illness and starvation, the third could be linked to predisposing factors for AN; however, inference from DTI parameters on actual histopathology and pathophysiology is challenging and subject of ongoing research (Jones et al., 2013).

### 4.1 Comparing acute changes in WM microstructure in adolescents with previous findings in adults

The results of increased FA and decreased MD in adolescents with AN are surprising when comparing with previous DTI studies on adult patients with AN which mainly reported regionally decreased FA and increased MD (Frieling et al., 2012, Hayes et al., 2015, Kazlouski et al., 2011, Nagahara et al., 2014, Via et al., 2014). Differences between our findings in adolescents with AN and those of adults could have developmental or time-related origins. A different mechanism could potentially dominate in the still-developing brain of adolescents compared to adults. The developing brain might be especially susceptible to metabolic challenges following starvation. Previous studies have demonstrated WM brain lesions in preterm infants, toddlers, children and adolescents to be age-specific and relatively pathogen-independent. Those structures that were currently under development were the most susceptible to damage (Gibson and Petersen, 1991). Thus, the typically late-developing association fibers in the anterior cingulate and prefrontal areas would be primarily targeted by starvation in adolescence, consistent with our and previous findings of fronto-cingulo-parietal alterations in AN (Frank et al., 2013, Kazlouski et al., 2011, Yau et al., 2013). In these late-developing areas, oligodendrocytes tend to support myelin sheaths of many more axons compared with those areas in the brain that mature early (Kochunov et al., 2011). Furthermore, oligodendrocytes in adults produce more and shorter internodes and thinner myelin sheaths than those of younger individuals (El Waly et al., 2014), potentially changing the susceptibility of axons and myelin to metabolic stress as a function of age.

Additionally, this difference between adolescents and adults with AN could be influenced by chronicity as adult patients generally tend to have been ill for a longer time than adolescents (e.g. 4.9 years (Nagahara et al., 2014) or 6.5 years (Via et al., 2014) compared with 1.2 years in our study). Rather, (sub-)acute changes in recently ill adolescents could be accompanied by (transient) FA increase, whereas more chronically ill adults may exhibit FA decreases typically interpreted as WM integrity disruption (Via et al., 2014): The correlation of increased FA at admission with higher rate of weight loss might point to an acute process, which would support this hypothesis. However, we did not observe a direct correlation between illness duration and increase of FA at admission in adolescents.

Conceivably, the partial reversibility of FA increases at discharge in our exploratory longitudinal analysis would support a rather acute and reversible pathomechanism. This could also help to explain the partial differences in our findings compared to the first DTI-study with adolescent AN patients (Frank et al., 2013). The patients in their study were re-alimented rather quickly, showing no significant reduction in overall GM or WM volume at the time of measurement. Potentially, also their FA values had partially decreased again at this point in treatment leaving only some areas with significant FA-increases and revealing some with decreases.

### 4.2 Preexisting brain alterations vs. illness consequences

The finding of increased FA could also be explained by preexisting WM alterations in patients with AN as it has previously been proposed by Yau et al. (Yau et al., 2013). However, at least partial starvation-related consequences as mentioned above seem to be more likely in our patient group. First, the diffusivity changes were widely distributed in the brain and were not limited to areas typically implicated in harm-avoidance or perfectionism as suggested by Yau et al. More likely, a similar trend of increased FA and reduced MD was found for the entire WM skeleton, pointing to a more global effect. Second, the increase in FA in our patients was especially prominent after rapid weight loss prior to admission, thus linking it to acute starvation effects rather than predisposing factors. Third, in our exploratory longitudinal study the microstructural changes observed during acute starvation at admission partly remitted after an average of four months of weight rehabilitation therapy. If the changes in the brain were mostly preexisting and correlated with character traits or other predisposing factors, the alterations would not be expected to respond to weight recovery.

### 4.3 Potential mechanisms of microstructural changes

A (sub-)acute temporary increase in FA and decreases in MD and RD as a consequence of starvation could be explained by a decrease of tissue-volume surrounding the axons, an increase in axonal diameter, a change in membrane permeability and an increase in myelination among other mechanisms (Jones et al., 2013).

A reduction in peri-axonal space has previously been found to cause increased FA and reduced RD in theoretical models (Ford et al., 1998) and would be in line with the often documented loss of overall WM volume in patients with AN (Seitz et al., 2014). Myelin-producing oligodendrocytes as well as astrocytes in the surrounding tissue could be affected by starvation and energy-deficiency, causing “cell shrinkage” and even cell death of these neuron-supporting cells (El Waly et al., 2014). This process might be particularly prominent in the developing brain (Gibson and Petersen, 1991). Thus far, there has been a lack of conclusive studies. The reduction in surrounding tissue could also be a consequence of dehydration, which could in turn cause a loss of extra-cellular water. However, in our study urine specific gravity was not increased in patients and did not correlate with any of the average diffusion parameters.

Increased axon and myelin sheath diameter could result from axonal or myelin sheath swelling following metabolic compromise in starvation (Beaulieu, 2002). Increased FA due to decreased RD has been found in humans during the acute phase of ischemia (Alexander et al., 2007) and following recent mild traumatic brain injury in adult (Bazarian et al., 2007) and adolescent patients (Chu et al., 2010). Notably, for brain injury patients initially increased FA changed over time and was reduced in the chronic phase. This corresponds to our findings and that of adult AN patients (Chu et al., 2010).

### 4.4 Limitations

The use of two different scan protocols might have influenced our results, especially the regression analysis of FA at admission with a strong contribution of the protocol to the overall model fitting. To counter this effect, we corrected all analyses for protocol type and had roughly evenly distributed groups of AN and TD measured with each protocol. Furthermore, our analysis reproduced similar findings of increased FA and reduced MD and RD even when limiting the analysis to those patients scanned with only one protocol. Using SPM as a second analysis protocol confirmed the direction of the results, albeit only on a more liberal significance level, potentially due to smaller method sensitivity. The results of our exploratory longitudinal study are obviously limited by the small sample size. Furthermore, longitudinal follow-up was limited to short-term weight gain.

### 4.5 Conclusion and clinical implications

Our findings in adolescents with AN at admission show that increased FA and micro-structural WM changes are associated with starvation and acute weight loss. In our exploratory longitudinal study, FA partially declined with short-term weight restoration, potentially pointing to a transient character of this increase.

Future studies with larger sample sizes and longer follow-up periods are needed to characterize the full time-course of WM changes in adolescents with AN, their potential for prediction of the clinical course of patients and whether early therapeutic interventions and rapid weight restoration can prevent acute and chronic WM damage.

The following are the supplementary data related to this article.

Supplementary material. Image 1

## Figures and Tables

**Table 1:** Overview over clinical parameters for AN patients and TD controls.

Variable	AN admission n = 22		AN discharge n = 9		Typically developing n = 21	
Mean	SD	Mean	SD	Mean	SD
Age (years)	15.03	1.60	14.76	2.30	15.17	1.28
BMI (kg/m^2^)	15.36	1.08	17.45	1.43	20.34	2.59
BMI-SDS (kg/m^2^)	− 2.41	0.72	− 1.07	0.32	− 0.04	0.84
BMI-percentile	1.8	3.13			49.79	26.68
Illness duration prior to admission (weeks)	57.80	62.42				
Weight loss prior to admission (kg)	11.31	5.98				
Rate of weight loss (kg/week)	0.34	0.27				
Rate of weight loss (BMI-SDS/week)	0.07	0.04				
Urinary specific gravity	1013.8	7.1	1015.0	3.5		
Therapy duration (weeks)			20.38	6.72		
Weight gain during therapy (kg)			8.03	2.86		
Rate of weight gain during therapy (kg/week)			0.40	0.12		
Rate of weight gain during therapy (BMI-SDS/week)			0.09	0.04		

AN — anorexia nervosa.

BMI-SDS — standardized body mass index.

SD — standard deviation.

TD — typically developing controls.

**Table 2:** Multiple linear regression for FA_skeleton at admission and discharge.

	Beta	p-Value
*FA_skeleton__admission_ (N = 17): total variance: R^2^ = 0.783, R^2^_corr_ 0.692, F = 8.651, p < 0.001*		
Age__admission_	0.130	0.450
BMI-SDS__admission_	0.215	0.186
Illness duration (weeks)	0.427	0.112
Rate of weight loss (BMI-SDS per week)	0.537	0.044
Protocol	0.999	0.000

*FA_skeleton__discharge_ (N = 9): total variance: R^2^ = 0.953, R^2^_corr_ 0.906, F = 20.287, p < 0.006*		
Age__discharge_	0.383	0.031
BMI-SDS__discharge_	0.747	0.004
Therapy duration (weeks)	− 0.814	0.011
WeightGain_BMI_SDS	0.249	0.274

BMI-SDS — standardized body mass index.

FA — fractional anisotropy.

Protocol — protocol type used.
