# Supplementary material for: White matter microstructural changes in adolescent anorexia nervosa including an exploratory longitudinal study
Source: Neuroimage Clin. 2016 Apr 12;11:614–21. doi: 10.1016/j.nicl.2016.04.002 (PMC4857215; doi:10.1016/j.nicl.2016.04.002)
Supplement: Supplementary file 1 — Supplementary material. [file mmc1.pdf]

Supplementary table 1: Alternative method (SPM) results comparing FA for adolescent AN at admission vs. TD corrected for age and protocol (cf. supplementary figure 5)

FA comparison TD versus $AN_{admission}$

Location (mm mm mm)			k_E	T (Peak level)	p_{uncorr} (Cluster level)
-22	-10	-4	35	4.56	0.152
54	-26	32	21	4.45	0.262
-48	6	18	14	3.76	0.360
-38	16	8	2	3.45	0.757
26	0	18	2	3.41	0.757
-26	-6	34	2	3.40	0.757
14	32	38	2	3.37	0.757
46	-46	50	1	3.35	0.839

FA comparison $AN_{admission}$ versus TD

Location (mm mm mm)			k_E	T (Peak level)	p_{uncorr} (Cluster level)
4	-14	-26	46	4.72	0.105
-8	-14	-30		3.38	
44	48	2	10	4.47	0.442
-46	-34	58	28	4.42	0.198
8	-94	16	132	4.42	0.010
0	-90	16		4.14	
-28	-90	-14	129	4.39	0.011
-18	-90	-14		3.82	
-36	8	58	7	4.38	0.526
60	6	22	7	4.35	0.526
10	-86	-12	46	4.23	0.105
-44	-84	-6	16	4.23	0.328
18	-14	44	31	4.13	0.176
-42	-86	6	9	4.03	0.467
38	48	-14	8	3.95	0.495
-30	12	4	7	3.95	0.526
32	-12	68	4	3.93	0.642
46	-34	8	48	3.90	0.098

AN – anorexia nervosa

FA – fractional anisotropy

k_E – number of voxels

p_{uncorr} – uncorrected p-value

SPM – Statistical Parametric Mapping

T – T-value

TD – typically developing controls

Supplementary material:

TBSS is an approach, which is developed for the analysis of DTI data to maximize alignment precision. A potential bias towards the brain regions with the regionally highest FA values might obscure changes in peripheral regions (Abe et al., 2010). SPM-based analyses were conducted to validate the results of our TBSS analyses. SPM-based analyses are not expected to show this bias, however, at the cost of reduced alignment precision.

To corroborate our TBSS-results, we chose to reanalyze the FA maps using Statistical Parametric Mapping 8 (SPM8) based on MATLAB 2013A. On the non-diffusion weighted B0 images of each data set, we utilized Unified Segmentation (Ashburner and Friston, 2005). The resulting deformation fields were applied to individual FA maps in order to transform them into MNI space. FA maps were smoothed with a 8 mm kernel. Using a two-sample t-test, pre- and post-ECT scans were compared. TFCE was used as implied in the TFCE toolbox v73 (http://dbm.neuro.uni-jena.de/tfce/) for SPM. For the results, the threshold was at $p(TFCE) < 0.001$.

Abe, O., Takao, H., Gonoi, W., Sasaki, H., Murakami, M., Kabasawa, H., Kawaguchi, H., Goto, M., Yamada, H., Yamasue, H., Kasai, K., Aoki, S., Ohtomo, K., 2010. Voxel-based analysis of the diffusion tensor. Neuroradiology 52, 699–710. doi:10.1007/s00234-010-0716-3

Ashburner, J., Friston, K.J., 2005. Unified segmentation. NeuroImage 26, 839–851. doi:10.1016/j.neuroimage.2005.02.018
